# Study on the Characteristics of Walnut Shell/Co-PES/Co-PA Powder Produced by Selective Laser Sintering

**DOI:** 10.3390/ma11050784

**Published:** 2018-05-11

**Authors:** Yueqiang Yu, Yanling Guo, Ting Jiang, Jian Li, Kaiyi Jiang, Hui Zhang, Yu Zhuang

**Affiliations:** 1 College of Mechanical and Electrical Engineering, Northeast Forestry University, Harbin 150040, China; yuyaoqiang.1228@163.com (Y.Y.); jiangting1112@163.com (T.J.); lijian499@163.com (J.L.); zh1226419340@163.com (H.Z.); 15776628158@163.com (Y.Z.); 2 Research and Development Center of 3D Printing Material and Technology, Northeast Forestry University, Harbin 150040, China; 3 College of Engineering and Technology, Northeast Forestry University, Harbin 150040, China; jevons007@163.com

**Keywords:** three-dimensional printing, selective laser sintering, agricultural and forestry wastes, walnut shell, polymer

## Abstract

Agricultural and forestry wastes are used as materials for selective laser sintering (SLS) to alleviate resource shortage, reduce the pollution of the environment, lower the cost of materials, and improve the accuracy of parts produced by SLS. However, the mechanical properties of wood–plastic parts are poor, and thus they cannot be applied widely. In order to improve the mechanical properties of wood–plastic parts, a new type of walnut shell polymer composite (WSPC) was prepared by a polymer mixing method and was used to produce parts via SLS. Additionally, the dimensional accuracy, morphologies, density, and mechanical properties of the WSPC parts were studied. The results showed that the addition of a small amount of copolyamide (Co-PA) powder could effectively improve the mechanical properties and decrease the density of the WSPC parts. By increasing the amount of Co-PA powder and decreasing that of copolyester (Co-PES) powder, the mechanical properties first increased, then decreased, and finally increased again; in addition, the density first decreased then increased. By increasing the preheating temperature, the mechanical properties and density of the WSPC parts were enhanced.

## 1. Introduction

Three-dimensional printing (3D printing), commonly known as additive manufacturing (AM) or rapid prototyping (RP), is an advanced manufacturing technology. On the basis of 3D digital model files and by using materials for selective laser sintering (SLS) mainly consisting of metals, ceramics, polymers, and their corresponding composites, 3D entities re fabricated by repeatedly depositing thin layers [1]. This technology mainly includes stereo lithography appearance (SLA), fused deposition modeling (FDM), SLS, laminated object manufacturing (LOM), and polyjet (PJ) [2]. SLS is a 3D printing technique which was introduced by Deckard [3]. SLS has some advantages over other 3D printing technologies, such as it does not need support during manufacturing, reuses materials, and presents a high accuracy of parts. Thus, SLS has been widely applied in various industries, such as automobile manufacturing, aerospace, casting, and medical industries [4,5,6,7].

With the development of SLS, the materials used in SLS play a decisive role in the quality of the final parts. In recent years, the materials most used for SLS are mainly metal, ceramic, polymer, and their corresponding composites [8,9,10,11,12,13,14,15,16,17]. Wood–plastic composites (WPC) parts produced by the conventional process have a relatively simple structure. More complex molds are needed to manufacture complex parts, which lead to a more complex manufacturing process, high production costs, and long production cycles. However, the SLS technology has a short production cycle and a high production precision and can produce parts with complex structures. Additionally, because of the demand of diversity and cost saving for SLS materials, authors developed sustainable, low-cost, and environmentally friendly WPC as new materials for SLS. After studying the sintering mechanism and sintering process of WPC, parts with small warping deformation and high dimensional accuracy were fabricated, consisting of wood powder composites [18,19,20], rice husk powder composites [21], bamboo powder composites [22], and walnut shell powder composites [23]. The formed parts were applied as new product prototypes for wood molds, investment castings, medical models, and handicrafts. Although SLS can produce WPC molded parts with complex structure and high precision, the mechanical properties of these formed parts were weak, probably because of the characteristic of polymers. The mechanical properties of the formed parts were improved by post-processing; however, good results were difficult to achieve because of the complex process of post-processing [24,25]. Therefore, it is essential to study the influence of the characteristics of the employed polymers on the quality of the produced WPC parts.

Compared to copolyester (Co-PES) powder, copolyamide (Co-PA) powder has advantages, such as low melt viscosity and strong bond strength. The low melt viscosity reduces the internal porosity of the formed parts, and the strong bond strength can improve the strength of the interface between the matrix and the filler, resulting in better mechanical properties of the formed parts. Therefore, in this research, walnut shell powder was used as a filler, and Co-PES powder and Co-PA powder were used as a matrix to study the sintering mechanism of walnut shell composites (WSPC) powder. Furthermore, the dimensional accuracy, density, and mechanical properties of the WSPC parts were assessed.

## 2. Materials and Methods

### 2.1. Experimental Materials

**Figure 1:**
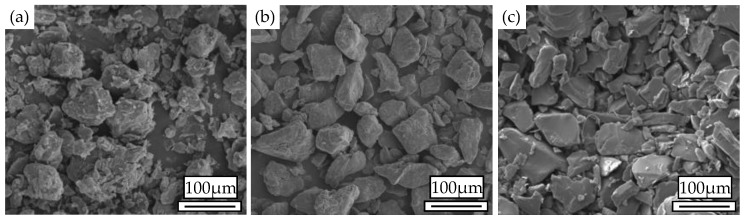
Morphologies of the powder particles: (**a**) walnut shell powder (WSP); (**b**) copolyester (Co-PES) powder; (**c**) copolyamide (Co-PA) powder.

Walnut shell powder (particle diameter range 58–96 µm and apparent density 0.48 g/cm^3^, approximately spherical porous particles) was obtained from Ding Sheng Corundum Abrasives Ltd. (Gongyi, China) and is shown in Figure 1a. Co-PES powder (particle diameter range 0–58 µm, apparent density 0.7 g/cm^3^, melt flow rate 30 g/10 min at 160 °C, viscosity 350 Pa·s at 160 °C, smooth-surfaced white block particles) was provided by Shanghai Tiannian Material Technology Ltd. (Shanghai, China) and is shown in Figure 1b. Co-PA powder (particle diameter range 0 µm–80 µm, apparent density 0.49 g/cm^3^, melt flow rate 35 g/10 min at 160 °C, viscosity 350 Pa·s at 160 °C, smooth-surfaced white block particles) was provided by Shanghai Tiannian Material Technology Ltd. (Shanghai, China) and is shown in Figure 1c. The light stabilizers (density 1.18 g/cm^3^, melting point 80 °C) was purchased from Zhenhai Jianghua Chemical Industry Ltd. (Ningbo, China), and the lubricant (zinc stearate density 1.095 g/cm^3^, melting point 125 °C) was obtained from Tianjin Guangfu Fine Research Institution (Tianjin, China).

### 2.2. Preparation of WSPC Powder

The WSPC powder mainly consisted of walnut shell powder, Co-PES powder, Co-PA powder and micro-additives. The waste walnut shell was processed by crushing, rotating, polishing, steaming, washing, and sieving, to obtain yellow-brown superficial porous particles. The Co-PES powder consists of white copolyester particles and was obtained from 1,2-butanediol, isophthalic acid, dimethyl terephthalate, and auxiliary additives through kettle reaction, cooling, dehydration, cryogenic pulverizing, and sieving. The Co-PA powder consists of white copolyamide powder particles and was obtained mainly by copolymerization of polyamide 66 and polyamide 1010 at a certain temperature, cooling, dehydration, cryogenic pulverizing, and sieving. The micro-additives used mainly consisted of small amounts of light stabilizers and lubricants, which improved the laser sintering performance and the mixing efficiency.

**Figure 2:**
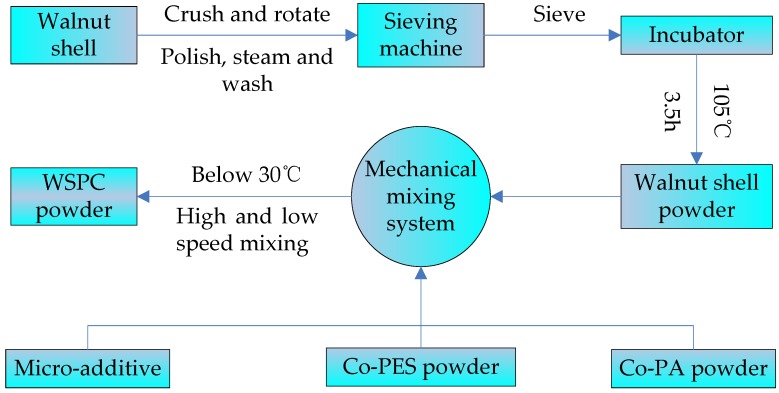
The process flow chart for the preparation of the WSPC powder.

The walnut shell powder was dehydrated for 3.5 h in an incubator (Longyuan Technology Ltd., Beijing, China) at 105 °C. During dehydration, the walnut shell powder was weighed at 1 h intervals until the mass became constant. Then, the dried walnut shell powder was mixed with Co-PES powder and Co-PA powder in different ratios (Table 1), using an SHR50A high-speed mixer from Hongji Machinery Ltd. (Zhangjiagang, China). To obtain the powder particles of the preferred dispersity and structural characteristics, the light stabilizers and lubricants were added during the mixing process. The powder was mixed for 15 min below 30 °C at low speed and then 5 min at high speed to keep the powder homogeneous. The mixed powder was taken out from the high-speed mixer and cooled to room temperature to obtain the WSPC powder for SLS. The preparation process of the WSPC powder is shown in Figure 2.

### 2.3. Equipment and Process Principle of SLS

**Figure 3:**
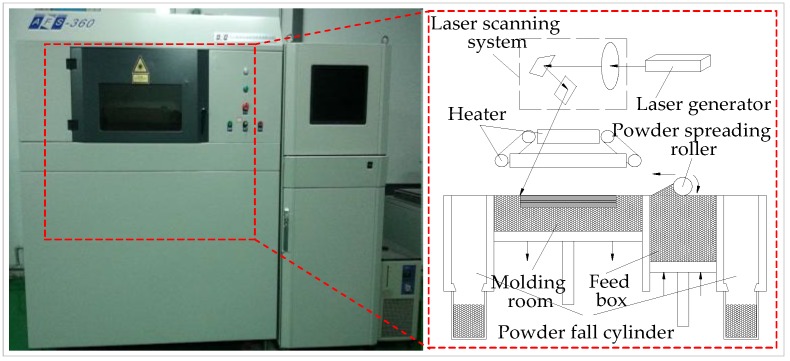
Equipment for selective laser sintering (SLS) and schematic diagram of the process.

The SLS experiments were conducted using an AFS-360 rapid prototyping equipment (Longyuan Technology Ltd., Beijing, China) equipped with a CO_2_ laser generator (wavelength of 10.6 μm and laser power of 55 W), a laser scanning system, a heat control system, a powder spreading system, and a working cylinder. The 3D objects were fabricated from a computer-aided design model by repeatedly depositing thin layers of fusible powder and selectively sintering each layer to the next with a modulated laser beam, a that is, the powder was spread by a powder spreading roller on the top surface of the molding room. The powder under the laser beam was fused and coalesced with the preceding layer, in a repeated fashion. The equipment for SLS and a schematic diagram of the process are shown in Figure 3.

**Figure 4:**
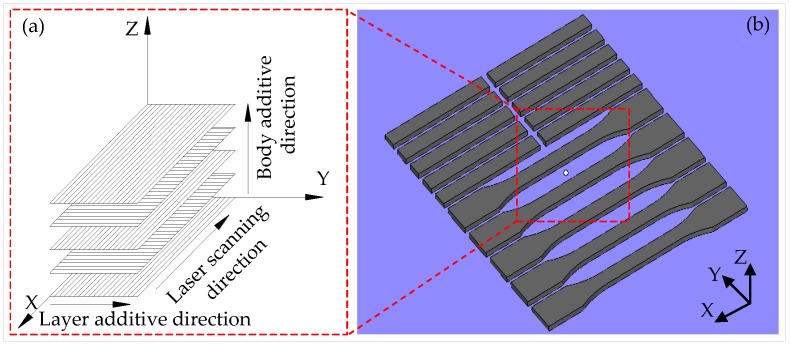
Sintering method: (**a**) additive direction and (**b**) placement of the sample.

To avoid thermal interference of the two samples during the laser sintering process, the distance between adjacent samples was set to 5 mm. The sample was placed horizontally and paralleled to the *X* axis of the equipment. To ensure that the sample was heated evenly, the sample was fixed in the middle of the sintering zone. The sintering method and the placement of the sample are shown in Figure 4. The processing parameters for SLS are shown in Table 2.

### 2.4. Characterization and Test

Differential scanning calorimetry (DSC): The Co-PES powder and Co-PA powder in the WSPC powder were analyzed using a Pyris Diamond differential scanning calorimeter from Perkin Elmer company (Waltham, Massachusetts, MA, USA). The testing parameters were as follows: the mass of the Co-PES powder and Co-PA powder was 5 mg; the heating rate was 10 °C/min; the testing temperature range was 40–200 °C. DSC curves of the Co-PES powder and Co-PA powder were obtained.

Scanning electronic microscopy (SEM): The walnut shell powder, Co-PES powder, Co-PA powder and cross sections of WSPC bending parts were first sputtered with gold because the specimens were non-conductive. Then, they were scanned using a FEI Quanta200 SEM produced by the Dutch company (Amsterdam, The Netherlands). The powder and various cross sections of the specimens were observed. A SEM diagram of the morphologies of the powder particles and of the inner morphologies of the WSPC parts was obtained.

Mechanical test: The mechanical performance of the WSPC parts was tested using a CMT5504 tensile testing machine (TMS System Ltd.) and a TCJ-4 impact testing machine by Jilin Province Taihe Ltd. (Changchun, China). The testing standards were as follows: The tensile strength was determined according to the ISO527-2 Standard, the crosshead speed was 5 mm/min, and the gauge length was 50 mm. The tree-point bending strength was determined according to the ISO178 Standard, the crosshead speed was 5 mm/min, and the span length was 64 mm. The U-notched impact strength was determined according to the ISO179-2 Standard, the pendulum impact power was 4 J, and the span length was 60 mm.

Density: The density of the WSPC part was measured using the Archimedes method. The mass of the part was measured using an electronic balance. An amount of water was injected into the graduated cylinder. Then, the WSPC part was placed in the graduated cylinder and was submerged in water with a fine needle. The density ρ of the WSPC part was calculated via Equation (1).

(1) $$\rho =\frac{W}{\left(V_{2}-V_{1}\right)}$$

where *W* is the mass of the part (g); *V*_1_ is the volume of water (mL); *V*_2_ is the total volume of the part and water (mL).

Dimensional precision: A cube specimen with dimensions of 20 mm × 20 mm × 20 mm was used in the precision analysis. The WSPC parts were fabricated by SLS according to the different process parameters indicated in Table 2. The actual dimensions of the WSPC parts were measured using a vernier caliper. The dimensional precision *δ* was calculated via Equation (2),

(2) $$\delta (\%)=(1-\frac{\left|L_{0}-L\right|}{L_{0}})$$

where *δ* represents the dimensional precision of the parts (%), *L*_0_ denotes the standard dimension of the parts (mm), and *L* indicates the actual dimension of the parts (mm). The dimensions of the WSPC parts in the X, Y, and Z directions were measured, and the dimensional precision of the WSPC parts in the X, Y, and Z direction was calculated.

## 3. Results and Discussion

### 3.1. Thermal Analysis of the WSPC Powder

**Figure 5:**
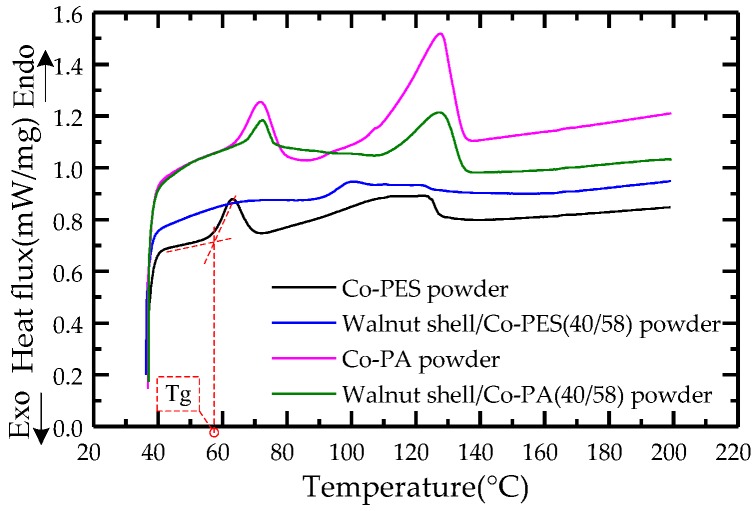
Differential scanning calorimetry (DSC) curves of the different powders.

In the process of SLS, the walnut shell powder used as a filler cannot melt and is considered the skeleton of the material; the Co-PES powder and Co-PA powder are considered the matrix of the material, as they can be melted by laser beam, thus forming a liquid phase, and wrap the walnut shell powder. The phase change of the polymers plays a main role in the process of SLS. Therefore, a thermal analysis of the different powders was conducted to determine the sintering window. The DSC curves of the different powders are shown in Figure 5. It is shown that the walnut shell had less influence on the polymer matrix, but the WSPC powder played an important role in the phase change of the polymer matrix. Therefore, it was essential to determine the glass transition temperature and agglomeration temperature of the matrix powder, which are different from those of crystalline polyamide 12 [26]. As shown in Figure 5, the glass transition temperatures of the Co-PES powder and Co-PA powder were 57.48 °C and 65.04 °C, respectively. From the experiments, it could be observed that the Co-PES powder and Co-PA powder agglomerated at 92 °C and 105 °C, respectively. Thus, the corresponding sintering windows were (57.48 °C, 92 °C) and (65.04 °C, 105 °C), respectively. Therefore, the sintering experiment could be conducted successfully by controlling the preheating temperature and processing temperature within those ranges.

### 3.2. Dimensional Precision

**Figure 6:**
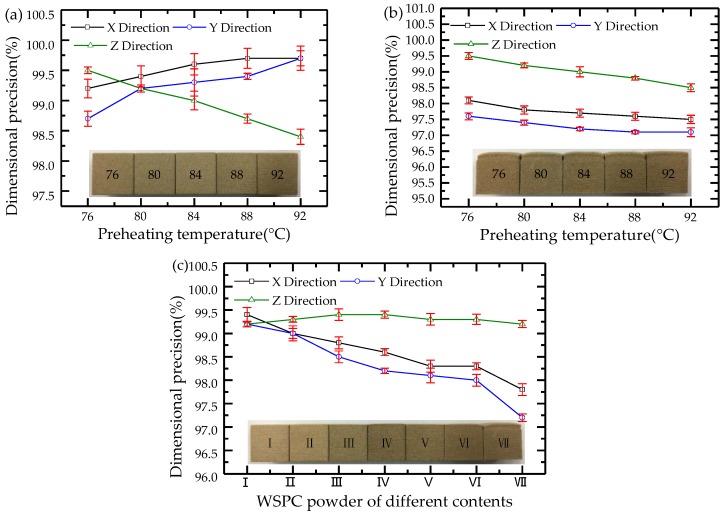
WSPC parts and change curves of dimensional precision of the WSPC parts: (**a**) walnut shell/Co-PES parts at different preheating temperatures; (**b**) walnut shell/Co-PA parts at different preheating temperatures; (**c**) WSPC parts with different powder contents.

The WSPC parts and the curves indicating the changes in dimensional accuracy of the WSPC parts are shown in Figure 6. Figure 6a shows the walnut shell/Co-PES parts at different preheating temperatures. It is shown that the shrinkage and warping deformation did not occur in the WSPC parts. By increasing the preheating temperature, the dimensional precision of the WSPC parts increases in the X and Y directions, but continuously decreased in the Z direction. Figure 6b shows the walnut shell/Co-PA parts at different preheating temperatures. The shrinkage and warping deformation of the WSPC parts were large. With increasing preheating temperatures, the dimensional precision of the WSPC parts gradually decreased in the X, Y, and Z direction. Figure 6c shows the WSPC parts with different powder contents. With an increase of the Co-PA powder, the shrinkage and warping deformation of the WSPC parts gradually increased. The dimensional precision of the WSPC parts continuously decreased in the X and Y directions, but almost remained constant in the Z direction. Therefore, these experiments revealed that the mixing of the Co-PES powder and Co-PA to prepare the WSPC powder improved the dimensional precision of the WSPC parts.

### 3.3. Morphologies

**Figure 7:**
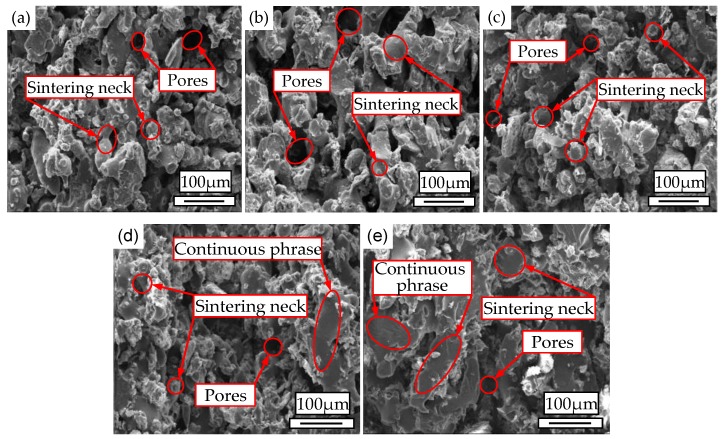
Scanning electronic microscopy (SEM) images of cross sections of the WSPC parts at the preheating temperature of 92 °C with walnut shell/Co-PES/Co-PA powder of (**a**) 40%/58%/0%; (**b**) 40%/50%/8%; (**c**) 40%/29%/29%; (**d**) 40%/18%/40%; (**e**) 40%/0%/58%.

The SEM images of cross sections of the WSPC parts at the preheating temperature of 92 °C are shown in Figure 7. Figure 7a shows that with a walnut shell/Co-PES/Co-PA powder of 40%/58%/0%, the size and quantity of the inner pores of the WSPC parts were large, and the sintering necks were small. This was mainly due to the low liquidity of the melted Co-PES powder. Figure 7b shows that with walnut shell/Co-PES/Co-PA powder of 40%/50%/8%, the size and quantity of the inner pores of the WSPC parts were small, and the sintering necks were large. The main reason is that when the Co-PES powder and Co-PA powder were both melted, the liquidity of the melted Co-PA powder was higher than that of the melted Co-PES powder. Figure 7c shows that with walnut shell/Co-PES/Co-PA powder of 40%/29%/29%, the size and quantity of the inner pores of the WSPC parts were large, and the sintering necks were small. The main reason is that the Co-PES powder and Co-PA powder could not absorb enough laser energy, thus the Co-PES powder was not fully melted, and the melt viscosity was high. Meanwhile, there was only a small amount of melted Co-PA powder because the Co-PA powder can be melted at a high temperature. Therefore, both powders could not melt fully [27]. Figure 7d shows that with walnut shell/Co-PES/Co-PA powder of 40%/18%/40%, a small amount of Co-PES powder led to an increase in the size and quantity of the inner pores of the WSPC parts, and the sintering necks were small. However, a small number of continuous phase occurred in the WSPC parts because the melted amount increased with the addition of the Co-PA powder. Figure 7e shows that with walnut shell/Co-PES/Co-PA powder of 40%/0%/58%, the size and quantity of the inner pores of the WSPC parts were small, and the sintering necks were large; a large continuous phase formed in the WSPC parts because of the higher liquidity of the melted Co-PA powder. Therefore, these experiments revealed that mixing the Co-PES powder and Co-PA powder to prepare the WSPC powder improved the compactness of the WSPC parts.

**Figure 8:**
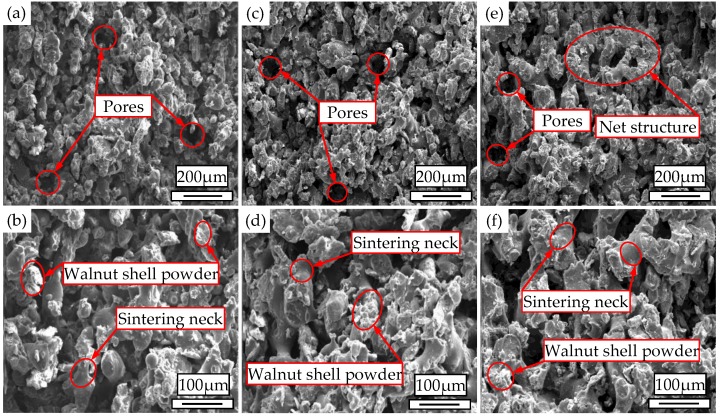
SEM images of cross-sections of the WSPC parts with walnut shell/Co-PES/Co-PA powder (40%/50%/8%) at (**a**,**b**) 76 °C; (**c**,**d**) 84 °C; and (**e**,**f**) 92 °C.

The SEM images of cross sections of the WSPC parts with walnut shell/Co-PES/Co-PA powder of 40%/50%/8% at different preheating temperatures are shown in Figure 8. Figure 8a,b show that the size and quantity of the inner pores of the WSPC parts were large, and the size and quantity of the sintering necks were small. The main reason is that at lower preheating temperatures, the laser energy absorbed by the Co-PES powder and Co-PA powder was less; thus, the liquid phase was less, and the walnut shell powder particles could not be fully wrapped. Figure 8c,d show that the size and quantity of the inner pores of the WSPC parts were small, the size of the sintering necks was small, and their quantity was large. The main reason is that by increasing the preheating temperature, the laser energy absorbed by the Co-PES powder and Co-PA powder increased; thus, the liquid phase increased, and the walnut shell powder particles could be fully wrapped. Figure 8e,f show that the size and quantity of the inner pores of the WSPC parts were small, and many large sintering necks formed a net structure. The main reason is that at even higher preheating temperatures, the laser energy absorbed by the Co-PES powder and Co-PA powder further increased; thus, the liquid phase increased, and the walnut shell powder particles could be fully wrapped.

### 3.4. Mechanical Properties

**Figure 9:**
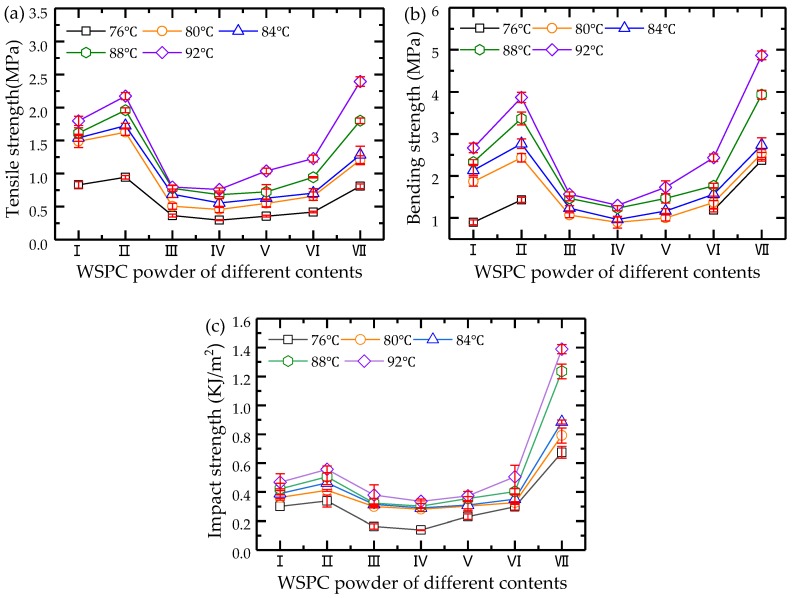
Mechanical properties changes of the WSPC parts: (**a**) tensile strength; (**b**) bending strength; (**c**) impact strength.

Figure 9 shows the curves indicating the changes in the mechanical properties of the WSPC parts. It is shown that the change tendencies of the tensile, bending, and impact strength of the WSPC parts were consistent. By increasing the amount of Co-PA powder and decreasing that of Co-PES powder, the mechanical properties of the WSPC parts increased first, then decreased, and later increased again. With an increase of the preheating temperature, the mechanical properties of the WSPC parts increased. The mechanical properties of the WSPC parts with walnut shell/Co-PES/Co-PA powder (40%/50%/8% and 40%/58%/0%) reached high values. The dimensional accuracy of the WSPC parts with walnut shell/Co-PES/Co-PA powder (40%/50%/8%) was the highest. Their tensile strength, bending strength, and impact strength were 2.174 MPa, 3.867 MPa, and 0.557 kJ/m^2^ at 92 °C, and increased by 20.9%, 45%, and 19%, compared to those of the WSPC parts with walnut shell/Co-PES/Co-PA powder (40%/50%/0%). The main reason is that with walnut shell/Co-PES/Co-PA powder (40%/58%/0%), the inner sintering necks of the WSPC parts were small, their interfacial strength was weak, and the mechanical properties were poor. With a small addition of Co-PA powder, the low melt viscosity produced larger sintering necks of WSPC parts with walnut shell/Co-PES/Co-PA powder of 40%/50%/8%. Therefore, their interfacial strength was enhanced, and the mechanical properties improved. By increasing the Co-PA powder, the small liquid phase produced smaller sintering necks of WSPC parts with walnut shell/Co-PES/Co-PA powders (40%/40%/18% and 40%/29%/29%), their interfacial strength was reduced, and the mechanical strength decreased. When the amount of Co-PA powder was higher than that of Co-PES powder, the large quantity of liquid corresponding to the melted Co-PA powder produced larger sintering necks and a continuous phase of WSPC parts with walnut shell/Co-PES/Co-PA powders (40%/18%/40%, 40%/8%/50% and 40%/0%/58%), with enhanced interfacial strength and improved mechanical properties.

### 3.5. Density of the WSPC Parts

**Figure 10:**
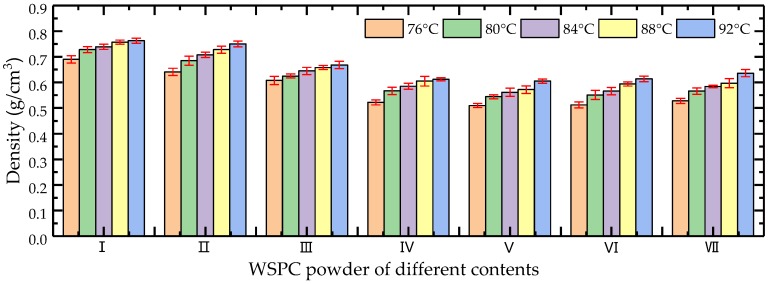
The bar graph of density changes of WSPC parts.

**Figure 11:**
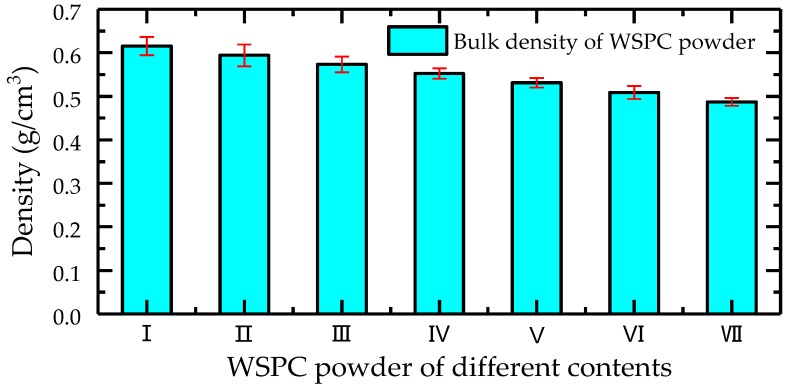
The bulk density of WSPC powder with different polymer powder contents.

The bar graph of the density changes of the WSPC parts is shown in Figure 10. The density of the WSPC parts first decreased, then increased when decreasing the amount of Co-PES powder and increasing the amount of Co-PA powder. The density of the WSPC parts gradually increased with increased preheating temperature. The main reason is that the bulk density of the Co-PES powder is larger than that of Co-PA powder when the amount of the Co-PES powder was larger than that of the Co-PA powder, and the bulk density of WSPC powder decreases as the increase of Co-PA powder (Figure 11). Additionally, the melting temperature of the Co-PES powder is lower than that of the Co-PA powder, resulting in less liquid phase. The size and quantity of the inner pores of the WSPC parts were large so that the density decreased. When the amount of the Co-PES powder was less than that of the Co-PA powder, the low melt viscosity of the melted Co-PA powder contributed to the small size and quantity of the inner pores of the WSPC parts. However, the melting temperature of the Co-PA powder is higher; thus, the density increased, rising slowly. With an increase of the preheating temperature, the laser energy absorbed by the WSPC powder increased, resulting in more liquid phase. The size and quantity of the inner pores of the WSPC parts decreased, and the density of the WSPC parts gradually increased.

## 4. Conclusions

The WSPC powder was prepared by a mechanical mixing method, and WSPC parts were fabricated by SLS technology. By analyzing the morphologies of cross sections of WSPC parts, the distribution of Co-PES powder and Co-PA powder in the WSPC parts appeared uneven. The dispersion phase depended on the addition of the Co-PES powder. The continuous phase depended on the addition of the Co-PA powder. In addition, the dispersion phase and continuous phase gradually increased with the increase of the preheating temperature. The dimensional precision, mechanical properties, and density were analyzed. Our results revealed that the dimensional precision of the walnut shell/Co-PES parts was higher than that of the walnut shell/Co-PA parts. However, the mechanical properties of the walnut shell/Co-PA parts were better than those of the walnut shell/Co-PES parts, and the density was lower. By blending the two polymers, the WSPC parts with walnut shell/Co-PES/Co-PA powder (40%/50%/8%) had higher dimensional precision and better mechanical properties, as well as lower density. Adding a small amount of Co-PA powder to the walnut shell/Co-PES matrix had a strengthening effect and made the WSPC parts lighter, resulting in WSPC parts with better mechanical properties and lighter weight. Therefore, it is feasible to fabricate WSPC parts displaying high accuracy, high mechanical properties, and light weight by controlling the process conditions.

## Figures and Tables

**Table 1:** Quantities of the ingredients in the walnut shell composites (WSPC) powder.

Serial Number	Walnut Shell Powder (vol %)	Co-PES Powder (vol %)	Co-PA Powder (vol %)	Micro-Additive (vol %)
Ⅰ	40	58	0	2
Ⅱ	40	50	8	2
Ⅲ	40	40	18	2
Ⅳ	40	29	29	2
Ⅴ	40	18	40	2
Ⅵ	40	8	50	2
Ⅶ	40	0	58	2

**Table 2:** Processing parameters of the WSPC powder for selective laser sintering (SLS).

Laser Power (W)	Scan Speed (mm/s)	Layer Thickness (mm)	Scan Spacing (mm)	Preheating Temperature (°C)					Processing Temperature (°C)
12	2000	0.15	0.2	76	80	84	88	92	75
